# Positive interpretation bias predicts well-being in medical interns

**DOI:** 10.3389/fpsyg.2014.00640

**Published:** 2014-06-24

**Authors:** Birgit Kleim, Hanna A. Thörn, Ulrike Ehlert

**Affiliations:** ^1^Department of Psychiatry, Psychotherapy and Psychosomatics, University of ZurichZurich, Switzerland; ^2^Department of Psychology, University of ZurichZurich, Switzerland; ^3^Department of Clinical Psychology and Psychotherapy, University of ZurichZurich, Switzerland

**Keywords:** depression, stress, resilience, cognitive predictors, interpretation bias

## Abstract

Cognitive theories of emotion posit that affective responses may be shaped by how individuals interpret emotion-eliciting situations. This study tested whether individual differences in interpretation bias (i.e., interpreting ambiguous scenarios in a more negative or positive manner) independently predict trait resilience and depression in medical interns. Interpretation bias and trait resilience scores were assessed in 47 interns prior to their first internship. Depressive symptoms were assessed twice during internship. Nearly half of the sample (42%) scored above the cut-off for mild depressive symptoms during internship, a significant rise compared to the initial assessment. Those with a more positive interpretation bias had higher trait resilience (β = 0.44, *p* = 0.004) and a 6-fold decreased depressive symptom risk during internship (*OR* = 6.41, *p* = 0.027). The predictive power of a positive interpretation bias for decreased depression symptoms held over and above initial depressive symptoms, demographics and trait reappraisal. Assessing positive interpretation bias may have practical utility for predicting future well-being in at risk-populations.

## Introduction

Cognitive theories of emotion posit that affective responses may be shaped by how individuals interpret emotion-eliciting situations. Beck's model of depression, for instance, highlights the tendency to interpret situations in a negatively biased manner as a key factor that may result in a negatively biased worldview and lead to depressed mood (Beck, 1976, 1987). Such a negative interpretation bias can be measured by means of responses to ambiguous scenarios (Butler and Mathews, 1983; Berna et al., 2011). For instance, a friend's muted response to receiving a present may be interpreted by one individual as a sign of contained excitement, whereas another individual may interpret the same reaction as disappointment and negative response.

Research has consistently confirmed that depressed individuals interpret information in a negatively biased manner. Such individuals choose negative interpretations more often than individuals without depressive symptoms; this finding has been confirmed with different ways of assessing interpretation biases, such as word sentence association paradigms (e.g., Hindash and Amir, 2012), color-naming interference, and interpretation of ambiguous situations (e.g., Nunn et al., 1997) or self-report questionnaires (e.g., Voncken et al., 2007). Individuals at risk for developing depression have also been shown to endorse a negative interpretation bias. Dearing and Gotlib (2009) found heightened negative interpretation of ambiguous scenarios in girls at risk for depression compared to girls at low risk. Initial evidence from prospective studies is in line with these findings and has confirmed the link between negative interpretation bias and the development of later depression. For instance, a study by Rude et al. (2002) assessed negative interpretation patterns using a scrambled sentences task and showed that a negative interpretation bias predicted subsequent depressive symptoms in undergraduate students.

While these studies highlight the adverse impact of a negative interpretation bias, there is little direct evidence for a potential protective role for a positive interpretation bias. So far, only a few studies have addressed this issue, demonstrating, for instance, a link between the ability to generate vivid positive imagery of future events and optimism (Blackwell et al., 2013), and between positive reinterpretation and decreased stress levels (Wood et al., 2007a,b). Interestingly, some research suggests that psychopathology, in this case anxiety, is associated with the absence of positive interpretation bias, rather than with the presence of negative interpretation bias (Hirsch and Mathews, 1997, 2000).

Based on these findings, it may be suggested (but has not been shown directly) that the tendency to interpret ambiguity in a positive manner may constitute a protective marker of well-being, particularly under conditions of stress. Individuals who tend to see the glass as perpetually half full rather than half-empty may experience more positive affect and be more stress-resilient. Stress resilience is defined as an individual's capacity to remain healthy in the face of stress and adversity and cope flexibly with these challenges (Bonanno, 2004). Resilient people have been characterized as more optimistic, cognitively flexible and endorsing positive coping styles; these characteristics are, in turn, associated with better mental health outcomes (Connor and Davidson, 2003). Resilient individuals may be less susceptible to depression and other stress-related conditions over time when faced with stress.

The present study examined interpretation bias in a group at risk of developing heightened depressive symptoms: medical students just about to undergo their first internship. Medical internship constitutes a well-established period of stress during which depressive symptoms increase in a significant number of interns (Sen et al., 2010; Guille et al., 2014). The study aimed to test whether individual differences in interpretation bias (i.e., interpreting ambiguous scenarios in a more negative or positive manner) independently predicted initial levels of stress resilience and risk for depression during internship. Interpretation bias was assessed using a well-established task, the Ambigous Scenario Task (Berna et al., 2011). For our study, we recruited medical interns and assessed their interpretation bias just before they commenced their internship and followed them up for 6 months during the internship indexing levels of depressive symptoms. Based on the literature above, we hypothesized that a baseline positive interpretation bias, indexed as the tendency to interpret ambiguous scenarios in a positive manner would be (i) associated with higher levels of trait resilience, hence constituting a potential buffer against stress and adversity, and (ii) predictive of well-being, as indexed by lower depression symptom risk as well as continuous depression symptoms during the course of the internship.

## Methods

### Participants

From a cohort of 200 medical students enrolled in their fifth year of medical training during the semester of recruitment, we consecutively recruited 48 students (20 male, 28 female, mean age = 24 years, *SD* = 1.99), see exclusion criteria below. Of these, 47 completed the Ambiguous Scenario Task. All participants had to commence their first internship in the following term. Exclusion criteria included presence of self-reported psychopathology, including depression and anxiety, scheduled internships in areas with projected low stressor exposure (e.g., dermatology). As the study was conducted in the framework of a not yet published fMRI study, additional standardized exclusion criteria applied regarding safe fMRI testing, e.g., no current medication and any medical or physical conditions, no metal in the participants' body, no tattoos etc.

From 94 students who initially expressed interest, a total of 40 individuals had to be excluded following these criteria, and 6 students canceled or did not appear to the laboratory session.

#### Study design and procedure

Participants were invited to take part in the study via study presentations in lectures given by the medical faculty for internship preparation, as well as an email sent out to all 5th year students. Those interested were screened via telephone for inclusion and exclusion criteria. During an individual laboratory session just before commencing their internship (T0, baseline measurement), they completed questionnaires as well as a computerized version of the Ambiguous Scenario task, AST-D. Three and six months later (T1, T2, follow-ups during internship), participants completed another questionnaire battery to assess their well-being, including depressive symptoms, as well as other internship-related variables. At the end of the study, participants were debriefed, and received reimbursement, i.e., 35 Swiss Francs for each hour spent in the laboratory. The local ethics review board, the cantonal ethical committee of Zurich, approved the study and all participants provided informed consent.

### Measures

#### Demographics

Participants filled in a number of items regarding demographic characteristics, such as age, sex, relationship status, as well as details of their prior medical experience (assessed at baseline, T0).

#### Prior trauma exposure

Exposure to prior trauma was indexed with a self-report questionnaire, which asked participants whether or not they had been exposed, at any point during their life, with 10 potential traumatic events, such as natural disasters, interpersonal violence, road traffic accidents, as well as an additional item where participants could add potential idiosyncratic personal events (assessed at baseline, T0).

#### Interpretation bias

Participants were presented with 24 individual ambiguous scenarios from the Ambiguous Scenario Task (AST-D, Berna et al., 2011), e.g., “It's New Year's Eve. You think about the year ahead of you.” They were instructed to form a mental image of each scenario and imagine it as if happening to them personally. They were then asked to rate pleasantness of the scenario, along with a rating of the vividness of each image. Scenarios were presented on the PC, one at a time, without time constraints, and participants entered their ratings using the numbers on the keyboard. Pleasantness ratings were given on a 7-point Likert scale anchored from 0 (“extremely unpleasant”) to 6 (“extremely pleasant”). Vividness ratings were also given on a 7-point Likert scale anchored from 0 “not vivid at all” to 6 “extremely vivid.” The AST-D had a Cronbach's α of 0.79 for pleasantness and 0.82 for vividness in the present sample, indicating good internal consistency. Pleasantness and vividness ratings were integrated into the logistic regression analyses as dichotomous scores, indexing whether individual biases were positive, (score above midpoint of the scale) or negative (score below midpoint of the scale) (pleasantness), vivid or not vivid (vividness). Linear regressions using the continuous score are also reported. The AST-D was assessed at baseline, T0.

#### Cognitive reappraisal

The Emotion Regulation Questionnaire (ERQ, Gross and John, 2003) assesses individual differences in the habitual use of emotion regulation. For the present study, the reappraisal subscale was used, indexing the individuals use of cognitive reappraisal with 6 items, e.g., “When I want to feel more positive emotion (such as joy or amusement), I change what I'm thinking about.” Items are answered on a 7-point Likert scale from 1 (“strongly disagree”) to 7 (“strongly agree”) and compiled to a sum score. Internal consistency of the subscale was good in the present sample, alpha was 0.89. The ERQ was assessed at baseline, T0.

#### Trait resilience

Participants completed the Connor-Davidson-Resilience Scale (CD-RISC, Connor and Davidson, 2003), a widely used self-report measure to index the ability to cope with stress and adversity. Respondents are asked to rate 25 items on a scale from 0 (“not true at all”) to 4 (“true nearly all the time”) and scores are calculated as a sum score with higher scores reflecting higher resilience. The scale has high internal consistency in non-clinical samples, with Cronbach's alphas of 0.76 and 0.89, respectively (Block and Kremen, 1996; Connor and Davidson, 2003). In the present sample Cronbach's alpha was 0.85 indicating high internal consistency. Trait resilience was assessed at baseline, T0.

#### Depression

The Patient Health Questionnaire (PHQ-9, Spitzer et al., 1999) was used to assess symptoms of depression at all three time points, T0, T1, T2. It is a self-administered version of the PRIME-MD instrument for common mental disorders. The PHQ-9 scores each of the 9 DSM-IV depression criteria as 0 (“not at all”) to 3 (“nearly every day”). The total PHQ-9 scores ranges from 0 to 27, with a cut-off score of 5 indicating mild depression (Kroenke et al., 2001), which was applied to the present data. The instrument is widely used in medical settings, with high validity and reliability scores (e.g., Kroenke et al., 2001). Cronbach's α in the present sample was 0.80 at the initial assessment, and 0.78 and 0.80 at the two follow-up time points, indicating good internal consistency.

### Data analyses

Participants with and without depression symptoms during their internship, i.e., those that met criteria for mild depression according to PHQ cut off-scores at either 3 or 6 months (or at both of these timepoints), were compared on demographic and internship-related variables using ANOVAs and χ^2^ Tests. We regressed trait resilience, depression symptom cut off as well as continuous depression scores on pleasantness and vividness mental imagery scores. We used linear regression analyses for continuous resilience and depression symptom scores at 3 and 6 months, and logistic regression analysis for dichotomous depression cut-off scores. Analyses were computed using initial depression symptom cut-off and trait emotional reappraisal scores as predictors, hence testing whether the predictive power of AST variables hold over and above these variables. Linear regression analyses were conducted with the continuous interpretation bias scores predicting continuous depression outcome at 3 and 6 months, respectively, as dependent variable. IBM PASW 20.0 was used for all analyses. A p value of *p* = 0.05 was considered significant.

## Results

### Trait resilience prior to internship and depression symptoms during internship

Trait resilience on the Connor Davidson Resilience Scale assessed prior to internship was high, *M* = 69.42, *SD* = 9.47, range: 47–88. Depressive symptoms increased significantly during the internship. Whereas 27% of medical students scored above the cut-off for mild depression prior to their internship, this score rose significantly to 42% during their internship, χ^2^ = 5.57, *p* = 0.018. Interns with and without increased depression symptoms during internship did not differ in terms of age, sex, exposure to previous trauma, or scoring above the depression cut-off prior to their internship, all *p*-values greater than 0.05. Correlations between study variables and descriptives can be found in Table 1.

**Table 1:** **Correlations among and descriptive statistics for key study variables (*N* = 47)**.

	**Mean (*SD*)**	**1.**	**2.**	**3.**	**4.**	**5.**	**6.**
1. Sex	0.58 (0.50)	–					
2. Age	24.44 (2.0)	−0.19	–				
3. Practical medical experience (years)	1.73 (0.45)	0.24	0.13	–			
4. Trait resilience	69.42 (9.47)	0.01	0.36^*^	−0.05	–		
5. Depression severity (T0)	3.48 (3.36)	0.05	−0.37^*^	−0.10	−0.48^**^	–	
6. Depression severity (T1)	4.44 (3.54)	0.34^*^	−0.40^**^	0.20	−0.35^*^	0.38^**^	–
7. Depression severity (T2)	4.35 (3.66)	0.27	0.27	0.15	−0.56^***^	0.39^**^	0.46^**^

For sex, 0 = male, 1 = female, trait resilience was assessed with the CD-RISC (Connor-Davidson Resilience Scale; Connor and Davidson, 2003); Depression severity was assessed with the PHQ-9 (Patient Health Questionnaire; Spitzer et al., 1999);

* p < 0.05,

** p < 0.01,

*** p < 0.001.

### Predicting trait resilience and depression with positive interpretation bias

Almost 60% (57.4%) of interns endorsed a tendency to interpret ambiguous scenarios in a positive manner, the remaining participants, 42.6%, interpreted them in an overall neutral or negative manner, overall *M* = 4.63, *SD* = 0.54. With respect to vividness, the majority of participants, 57%, described their imagery as vivid, overall *M* = 4.86 *SD* = 0.69. Initial depression symptoms were significantly associated with a negative interpretation bias, β = −0.45, *p* = 0.004, *R*^2^ = 0.25.

As predicted, trait resilience was associated with a positive interpretation bias, see Table 2. Individuals who tended to interpret emotional scenarios in a more positive manner reported higher trait resilience, β = 0.44, *p* < 0.001, *R*^2^ = 0.23. Vividness scores were not significantly related to resilience. Moreover, as shown in Table 3, the tendency to interpret ambiguous scenarios in a more positive manner was associated with a 6-fold decreased risk of depression symptoms during internship, *OR* = 6.25 (1.2–33.3). It was also associated with heightened depressive symptom severity at 6 months, β = −0.34, *p* = 0.027 (Table 4). The relationship between interpretation bias and depression held when initial depression cut-off and trait emotion regulation strategies, i.e., the tendency to reappraise, were controlled for. Vividness of mental imagery was not significantly related to depression symptoms during internship. Similar results were obtained using a continuous interpretation bias score, i.e., individuals who endorsed more positive interpretations of ambiguous scenarios also reported decreased depression symptoms at 6 months, when controlling for initial depression symptoms as well as trait emotion regulation (see Table 4, Model 2), overall model: *R*^2^ = 0.27, *F*_(4, 46)_ = 3.86, *p* = 0.009. Again, pleasantness was driving the effect in this analysis, β = −0.36, *p* = 0.023, indicating that those with more positive imagery reported less depression at 6 months, whilst vividness was not a significant predictor, β = 0.23, *p* = 0.139. Depression at 3 months was not significantly predicted by interpretation bias scores, β = −0.27, *p* = 0.110, overall model: *R*^2^ = 0.11, *F*_(3, 46)_ = 1.71, *p* = 0.179.

**Table 2:** **Positive interpretation bias predicts resilience cross-sectionally**.

	***R*^2^**	***p***	***B***	β	***p***
Overall model	0.23	0.003			
Pleasantness of imagery (AST)			8.21	0.44	0.004
Vividness of imagery (AST)			1.67	0.09	0.535

Dependent variable: trait resilience score (CD-RISC; Connor Davidson Resilience Scale; Connor and Davidson, 2003); AST, Ambiguous scenarios task (Berna et al., 2011); B, unstandardized Beta.

**Table 3:** **Positive interpretation bias prospectively predicts depression cut off during internship beyond initial depression and trait reappraisal**.

	**Nagelkerke *R*^2^**	***p***	**OR (95%CI)**	**Wald**	***p***
Step 1: Control variables	0.20	0.021			
Initial mild depression cut-off (PHQ-9)			5.01 (1.24–20.26)	5.11	0.024
Trait reappraisal (ERQ)			1.01 (0.98–1.04)	0.55	0.457
Step 2: Interpretation bias	0.35	0.007			
Initial mild depression cut-off (PHQ-9)			5.63 (1.01–31.04)	3.89	0.049
Trait reappraisal (ERQ)			1.02 (0.98–1.06)	0.76	0.384
Pleasantness of imagery (AST)			6.41 (1.22–33.33)	4.88	0.027
Vividness of imagery (AST)			3.79 (0.65–21.95)	2.21	0.137

Dependent variable: cut-off for depression symptoms during one of the two follow-up time points; PHQ-9, Personal health questionnaire (Spitzer et al., 1999); ERQ, Emotion regulation questionnaire (Gross and John, 2003); AST, Ambiguous scenarios task (Berna et al., 2011); predictor multicollinearity was within an acceptable range for all predictors, i.e., range of tolerance = 0.80–94.

**Table 4:** **Positive interpretation bias prospectively predicts depression symptom severity at 6 months during internship beyond initial depression and trait reappraisal using dichotomous as well as continuous bias scores**.

	***R*^2^**	***p***	***B***	β	***p***
**MODEL 1: DICHOTOMOUS INTERPRETATION BIAS SCORES**					
Step 1: Control variables	0.17	0.017			
Mild depression cut-off (PHQ-9)			3.05	0.40	<0.001
Trait reappraisal (ERQ)			0.001	0.03	0.822
Step 2: Interpretation bias	0.27	0.010			
Mild depression cut-off (PHQ-9)			2.85	0.39	0.007
Trait reappraisal (ERQ)			0.004	0.10	0.469
Pleasantness of imagery (AST)			−2.30	−0.34	0.027
Vividness of imagery (AST)			0.82	0.23	0.139
**MODEL 2: CONTINUOUS INTERPRETATION BIAS SCORES (STEP 1 IDENTICAL AS ABOVE)**					
Step 2: Interpretation bias	0.27	0.009			
Mild depression cut-off (PHQ-9)			2.74	0.36	0.012
Trait reappraisal (ERQ)			0.02	0.04	0.753
Pleasantness of imagery (AST)			−2.48	−0.36	0.023
Vividness of imagery (AST)			0.82	0.23	0.139

Dependent variable: continuous depression score at 6 months; PHQ-9, Personal health questionnaire (Spitzer et al., 1999); ERQ, Emotion regulation questionnaire (Gross and John, 2003); AST, Ambiguous scenarios task (Berna et al., 2011). Presented are analyses using the dichotomized AST score (Model 1), as well as the continuous AST score (Model 2).

## Discussion

We investigated the predictive power of a positive interpretation bias on resilience and prospective depressive symptoms in an at-risk population, medical students commencing their first internship. During this period, a considerable subgroup of individuals tends to react with stress and symptoms of depression (Sen et al., 2010; Guille et al., 2014). The significant rise in depression symptoms from the initial assessment to the internship period in our sample stands in line with these prior studies. Resilience, assessed initially, was high in the present sample compared to studies with similar samples (e.g., Peng et al., 2012). As hypothesized, a positive interpretation bias indexed just prior to the internship was (i) significantly associated with self-reported trait resilience, and (ii) predictive of a reduced depression risk during the course of the internship (the following 6 months) and predictive of depression severity at 6 months, even after controlling for initial depression and trait reappraisal. Depression severity at 3 months was not significantly predicted by interpretation bias. In accord with Berna and colleagues' results, we found that pleasantness of the imagined scenarios, rather than vividness of participants' imagery, significantly predicted resilience, reduced depression risk and depression severity at 6 months (Berna et al., 2011). Taken together, our results suggest that a positive interpretation bias is protective in the face of stress and adversity and may be a cognitive marker for well-being in populations at risk. With the advent of interpretation bias modification paradigms, positive interpretation bias could be a key factor in the context of developing prevention and early interventions efforts (Hertel and Mathews, 2011).

Prior studies have primarily focused on a negative interpretation bias, although some studies noted the absence of a positive interpretation bias in anxious populations and underscored the relevance of a positive interpretation bias (Hirsch and Mathews, 1997, 2000). Our results expand on these studies and suggest that a positive interpretation bias can be seen as a protective marker and may act as a buffer against stress and adversity by increasing resilience or decreasing depression risk. This finding is in line with recent efforts to investigate the mechanisms and pathways associated with resilience, including cognitive and emotional processes that affect the relationship between stress and resilience (e.g., Southwick and Charney, 2012; Wu et al., 2013). A positive interpretation bias may thus constitute one factor on the pathway to resilience. It is conceivable that a positive interpretation bias exerts its protective effects via the experience of positive emotions, for instance by contributing to participants' ability to achieve efficient emotion regulation. Resilient individuals have indeed been shown to use positive emotions to bounce back from stress and adversity (Tugade and Fredrickson, 2004). Future studies will have to determine, however, the precise mechanisms and orchestration between positive interpretation bias and other key factors such as genetic, developmental and neurobiological risk and protective factors.

In line with the association between positive interpretation bias and resilience is the finding that those who tend to interpret ambiguous personal situations in a positive manner had a 6-fold decreased self-reported depression risk during their internship. The magnitude of this reduction in depression symptoms in the subgroup endorsing a positive compared to a negative interpretation bias in our study appears remarkable and can be considered as a large effect compared to some other established risk and protective factors for depression (e.g., Hirschfeld and Weisman, 2002). Moreover, we found that this protective effect of positive interpretation bias was not attenuated when initial depression and trait reappraisal were adjusted, hence underlining the unique explanatory power of a positive interpretation bias. In contrast to our hypothesis, however, interpretation bias was not a significant predictor of depression severity at 3 months in our study. Depressive symptoms may fluctuate over time, possibly along variation in internship-related stressor exposure. Such affective instability and intense fluctuations in emotions in response to both pleasant and unpleasant events has indeed been observed in students (Thompson et al., 2011) and may account for this finding. Further research is necessary to corroborate the association between interpretation bias and reduced depression at early and later stages in populations exposed to stress and adversity.

Provided replicability of our findings, the present results have practical clinical implications. assessing positive interpretation bias may have practical utility for characterizing potentially resilient subgroups versus those at risk for depression and for predicting future well-being in at risk-populations. The results also point toward a benefit of training positive interpretation bias in the context of prevention programs. There is indeed accumulating evidence that interpretative biases can be experimentally induced using ambiguous scenarios (e.g., Tran et al., 2011). Recently developed cognitive bias modification (CBM) paradigms aim at changing individuals' biases toward a more positive or optimistic direction by providing standardized positive resolutions to ambiguous scenarios that participants are instructed to imagine vividly in front of their own eyes (e.g., Holmes et al., 2006; Hertel and Mathews, 2011). These CBM paradigms have the potential to induce symptom reduction in the context of dysphoria and depression (e.g., Blackwell and Holmes, 2010; Williams et al., 2013). The present results would advocate such CBM training toward positive interpretation bias as an important component of prevention and intervention efforts targeted toward those at risk for depression. Such training procedures, if developed, could helpfully complement interventions aimed at preventing depression and boosting resilience.

The present study is not without limitations. First, we focused on interpretation bias, whereas recent work has suggested that interpretation, attention and memory biases are likely to be closely associated and influence each other (e.g., Everaert et al., 2013), and should therefore be studied in concert. Moreover, certain conditions have been shown to moderate expression of an interpretation bias, for instance, self-reference of the scenarios (Wisco and Nolen-Hoeksema, 2010) or cognitive load (Rude et al., 2002). These factors should be considered in future studies. Second, the AST-D requires participants to imagine and describe internal processes, i.e., participants' specific imagery and their idiosyncratic interpretations of a given scenario. Whilst this approach is likely to be more valid than self-report questionnaires of trait interpretation biases, it is still subject to certain response formats, which should be further investigated. Third, the association between positive interpretation bias and resilience is cross-sectional and precludes any firm causal conclusion regarding temporal associations between the two. Further longitudinal studies with several assessments of both variables, as well as experimental approaches would be needed in order to make true causal claims. Fourth, we assessed depression symptoms with a self-report questionnaire and clinician-rated depression symptoms might be more valid and thus needed in future studies. Finally, our sample was rather small, hence restricting the power of our analyses. It also comprised a unique sample, medical students just before and during their medical internship, the results may thus not be transferable to other populations.

In sum, we showed that interpretation bias is associated with trait resilience and predicts depression symptom risk prospectively. These findings have important implications, suggesting that (i) a simple assessment of positive interpretation bias may have practical utility in predicting future well-being and depression in at risk-populations and (ii) in the future exploring the inclusion of modules of training toward a more positive interpretation bias into current therapies for depression or depression prevention programs may increase the effectiveness of these interventions.

### Conflict of interest statement

The authors declare that the research was conducted in the absence of any commercial or financial relationships that could be construed as a potential conflict of interest.
